# Integrating heterogeneous genomic data to accurately identify disease subtypes

**DOI:** 10.1186/s12920-015-0154-5

**Published:** 2015-11-20

**Authors:** Xianwen Ren, Hua Fu, Qi Jin

**Affiliations:** MOH Key Laboratory of Systems Biology of Pathogens, Institute of Pathogen Biology, Chinese Academy of Medical Sciences and Peking Union Medical College, Beijing, 100730 China

**Keywords:** DNA methylation, Gene expression, miRNA expression, Integration, Diagnosis, Prognosis, Cancer stratification

## Abstract

**Background:**

High-throughput biotechnologies have been widely used to characterize clinical samples from various perspectives e.g., epigenomics, genomics and transcriptomics. However, because of the heterogeneity of these technologies and their outputs, individual analysis of the various types of data is hard to create a comprehensive view of disease subtypes. Integrative methods are of pressing need.

**Methods:**

In this study, we evaluated the possible issues that hamper integrative analysis of the heterogeneous disease data types, and proposed iBFE, an effective and efficient computational method to subvert those issues from a feature extraction perspective.

**Results:**

Strict experiments on both simulated and real datasets demonstrated that iBFE can easily overcome issues caused by scale conflicts, noise conflicts, incompleteness of patient relationships, and conflicts between patient relationships, and that iBFE can effectively combine the merits of DNA methylation, mRNA expression and microRNA (miRNA) expression datasets to accurately identify disease subtypes of significantly different prognosis.

**Conclusions:**

iBFE is an effective and efficient method for integrative analysis of heterogeneous genomic data to accurately identify disease subtypes. The Matlab code of iBFE is freely available from http://zhangroup.aporc.org/iBFE.

**Electronic supplementary material:**

The online version of this article (doi:10.1186/s12920-015-0154-5) contains supplementary material, which is available to authorized users.

## Background

With the development of high-throughput genomic technologies, it has become easy and cost-effective to comprehensively characterize clinical samples by a wide range of genomic data, e.g., depicting cancer samples from epigenomic, genomic and transcriptomic perspectives. Large-scale efforts conducted by The Cancer Genome Atlas (TCGA) have already applied this strategy to study over 20 cancers from thousands of patients, with a large amount of epigenomic, genomic, transcriptomic and clinical data collected from the same patients [1–4]. While the availability of such a wealth of well-structured data makes the status of patients be characterized comprehensively and subtly, it also presents important challenges for the analysis methodology. Because of the great heterogeneity of technologies and biological data, individual analysis or simple concatenation of all the available datasets often cannot generate desired results [5]. Although independent analyses of single datasets were commonly adopted, the inconsistent conclusions underscore the necessity of unbiased integrative methods. Due to the exacerbated “curse of dimensionality” [6], i.e., the number of measures is greatly larger than the number of patients, direct concatenation may generate worse results. The currently developed integrative methods for analysis of multiple genomic data of the same patients can generally be classified into three groups [7, 8]. The first group of methods is based on matrix factorization [9–13]. The second group of methods is based on Bayesian models [14–16]. A major issue with the factorization and Bayesian approaches is that they generally require proper data preprocessing and normalization techniques. The computation of these approaches is also complicated. Recently, Wang et al. proposed a new type of integrative methods based on network fusion, which achieves the state-of-the-art performance regarding both accuracy and computational speed as demonstrated in [5]. However, it is still unknown what factors interfere with integrative analysis and what are the pitfalls of the current integrative analytical methodology while dissection of issues that interfere with integrative analysis and identification of alternative methods is essential for boosting the translation of advances of high-throughput genomic technologies to personalized medicine.

In this study, we explicitly interrogated factors that inhibit integrative analyses of multiple data types for both disease class discoveries and classifications [17]. By isolating those possible factors, we identified that the scales of measurement, the noise types and sizes, and the completeness and concordance of patient relationships in different data types are important issues that prevent integrative. And the currently available methods cannot overcome all the issues. Motivated by the great power of feature extraction methods for unbiased and unsupervised analyses in single datasets [18], we proposed a novel integrative approach Based on Feature Extraction (referred to iBFE below). Simulations suggested that iBFE can overcome all the issues identified in this study. Applications of iBFE to integrating the DNA methylation, mRNA expression and miRNA expression datasets of lung and kidney cancers produced by TCGA suggest that iBFE not only can successfully integrate the diverse data types but also can identify disease subtypes that have distinct survival profiles. Because iBFE is simple, flexible, unsupervised and unbiased, it is readily to extend to integrate more types of genomic datasets to improve the disease diagnosis and prognosis.

## Methods

### Overview of the iBFE method

The iBFE method is motivated by the observation that the accuracy of disease class discovery and classification can be significantly improved in the feature space extracted from the original data [18–20]. The pipeline of iBFE consists of three steps: i) extract features from individual type of datasets; ii) concatenate the extracted features; iii) extract new features from concatenated features. When the three steps were finished, the newly constructed features of patients can be used as inputs to do disease class discoveries and classifications by other algorithms e.g., *k*-means [21, 22] and support vector machines [23, 24].

First, iBFE uses Pearson and Spearman correlations to extract features from individual data types. Given a single dataset X_MxN_^(1)^, in which x_ij_^(1)^ represents the j-th variable of the i-th patient (i ranged from 1 to M, and j ranged from 1 to N), P_MxM_^(1)^ and S_MxM_^(1)^ are constructed from X^(1)^. P^(1)^ is the similarity matrix of patients constructed by Pearson correlation coefficients [25, 26], i.e., p_ab_^(1)^ is the Pearson correlation coefficient of x_a-_^(1)^ and x_b-_^(1)^. Here x_a-_^(1)^ and x_b-_^(1)^ represent values of all the variables of the a-th and b-th patients, respectively. Similar to P^(1)^, S^(1)^ is the similarity matrix of patients constructed by Spearman correlation coefficients [27, 28]. The advantage of Pearson correlation coefficients in feature extraction has been demonstrated and validated previously [18]. The introduction of Spearman correlation coefficients here is to employ its distribution-independent property, which is important for handling issues caused by scale and noise during integration. Both of Pearson correlation coefficients and Spearman correlation coefficients have values ranged from −1 to 1, which can provide consistent scales for different data types.

Given K types of datasets, in the second step, P^(k)^ and^(k)^, k = 1,…,K, are concatenated into Y_Mx2MK_, i.e., Y_Mx2MK_ = [P^(1)^S^(1)^ … P^(k)^S^(k)^ … P^(K)^S^(K)^], where the rows of Y represent patients while the columns of Y are the extracted features by Pearson correlation coefficients and Spearman correlation coefficients. Because P^(k)^ and S^(k)^ are naturally normalized to the region from −1 to 1, concatenation at this step will not suffer from issues encountered during direct concatenation of the original datasets.

In the third step, a new similarity matrix of patients Z_MxM_ is constructed by calculating the Pearson correlation coefficients of the rows of Y, i.e., z_ij_ is the Pearson correlation coefficient of y_i-_ and y_j-_, where y_i-_ and y_j-_ represent the i-th and j-th rows of Y, respectively. Z_MxM_ is the final features extracted by iBFE from the K types of original datasets. In practice, the original datasets generally consist of thousands of variables because thousands of genes are measured at the epigenomic, genomic and transcriptomic levels by high-throughput biotechnologies. By mapping the original datasets into feature space spanned by profiles of patient similarities, iBFE extracts the patterns embedding within patient relationships. Further, the calculation expense is also greatly reduced.

In summary, the algorithm of iBFE can be outlined as follows:

- Step I: calculate P(k) and S(k) for X(k), k = 1,…,K;
- Step II: construct Y = [P^(1)^ S^(1)^ … P^(k)^ S^(k)^ … P^(K)^ S^(K)^];
- Step III: construct Z by calculating the Pearson correlation coefficients of rows of Y.

Here we named the iBFE using both Pearson and Spearman correlation coefficients as iBFE_1_. To evaluate the performance of iBFE that only employs Pearson or Spearman correlation coefficients, we also constructed iBFE_2_ that only uses Pearson correlation coefficients and iBFE_3_ that only uses Spearman correlation coefficients.

### Simulating datasets that dissect possible issues interfering with integration

**Fig. 1:**
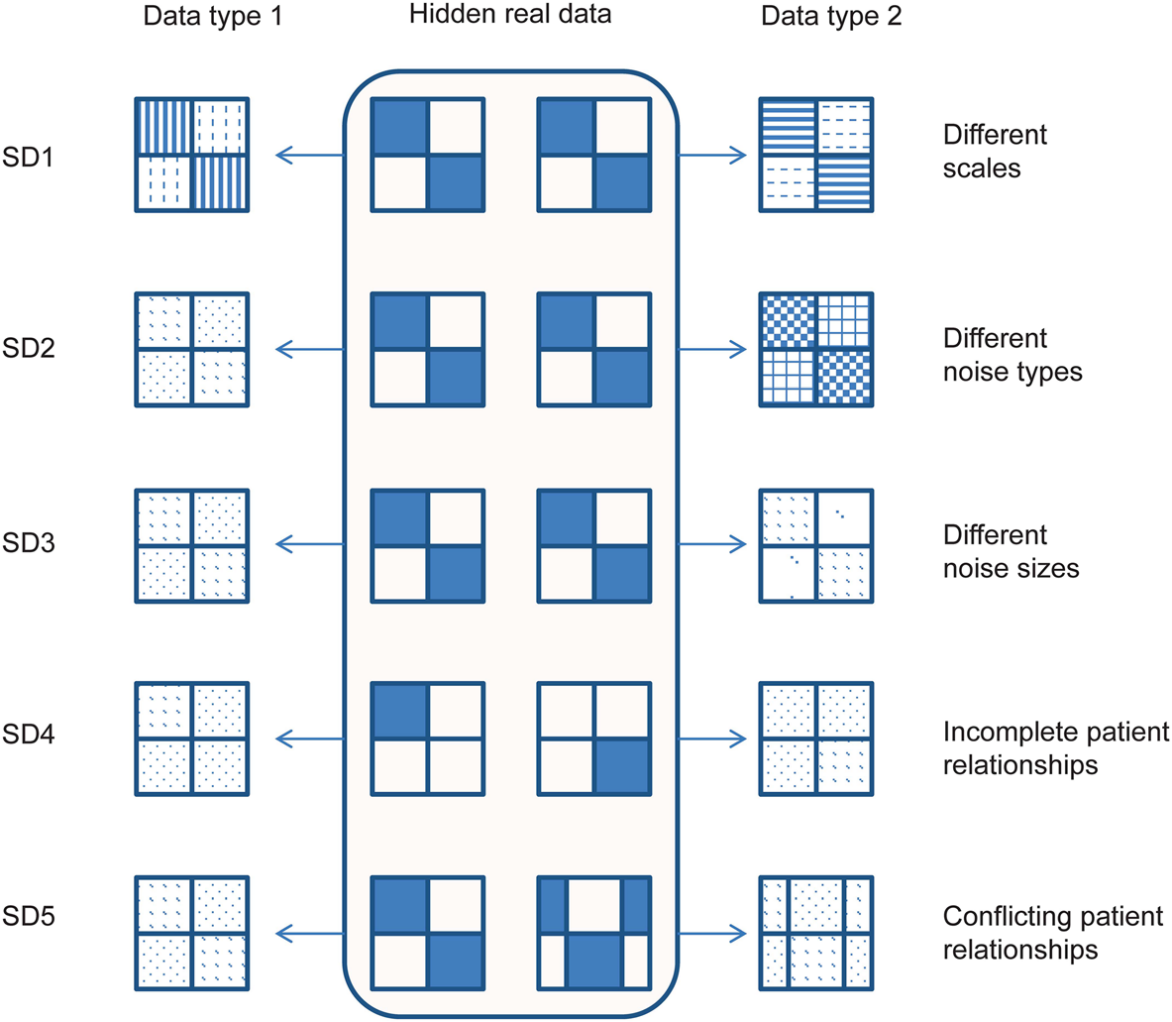
Graphic representations of the simulation process. A total of five simulated datasets were generated. Each dataset was simulated by firstly constructing two prototype data types and then adding noise (represented by the shadows in the figure with different shadow types representing different noise types). Simulated dataset 1 (SD1) simulated the impacts of different scales on the integrative analyses through scaling in different ways of the same prototype dataset adding the same type and size of noise. SD2 simulated the impacts of different types of noise on the integrative analyses through adding different types of noise to the same prototype dataset. SD3 simulated the impacts of different sizes of noise through adding different sizes but the same type of noise to the same prototype dataset. SD4 simulated the impacts of incompleteness of patient relationships through constructing partially clustered prototype datasets. SD5 simulated the impacts of conflicting patient relationships through constructing conflicting clustered prototype datasets

We evaluated the factors that may affect integration of different types of datasets for disease class discovery and classification by simulation. Because simulation can highlight one possible factor while controlling the influence of other factors, it provides an ideal tool to evaluate the impacts of single factors on integration although some simulations may be not quite realistic. According to our experience, we hypothesize that the following factors that may affect integrative analyses: i) scales of measurements in different datasets; ii) noise types of different datasets; iii) noise sizes; iv) completeness of patient relationships that is revealed by single datasets; v) concordance of patient relationships revealed by each dataset. To evaluate their roles during integrative analyses, we constructed five simulated datasets (Fig. 1).

By simulated dataset 1 (SD1), we evaluated the impacts of scale conflicts of measurements on integrative analyses. We simulated 100 patients that are characterized by 100 variables for simplicity. The first 50 patients belong to cluster 1, with the first 50 variables all one and the other 50 variables all zero. The second 50 patients belong to cluster 2, with the first 50 variables all zero and the other 50 variables all one. All the 100x100 measurements are disturbed by noise sampling from a standard normal distribution. We named this prototype data as data 0 (SD1-D0), the hidden real data. Two types of observed data are generated from SD1-D0. Type 1 of SD1 (SD1-T1) is constructed by transforming SD1-D0 to its q^th^ power, i.e., x_ij_^(SD1-T1)^ = (x_ij_^(SD1-D0)^)^q^. Type 2 of SD1 (SD1-T2) is constructed by x_ij_^(SD1-T2)^ = q^(x_ij_^(SD1-D0)^). Here q is a parameter to control the scale difference between the two data types. The power-law and exponential functions are used to simulate the issues caused by scales of different measurements.

By simulated dataset 2 (SD2), we evaluated the impacts of different noise types on integrative analyses. The prototype SD2-D0 is the same as SD1-D0 except that the noise is not added. The observed SD2-T1 and SD2-T2 are constructed based on SD2-D0 by adding noise sampled respectively from a normal distribution with means zero and standard deviation q and from a uniform distribution from zero to q, where q is the parameter to control the size of noise.

By simulated dataset 3 (SD3), the impacts of different noise sizes are evaluated. The prototype SD3-D0 is the same as SD2-D0. The observed SD3-T1 and SD3-T2 are constructed based on SD3-D0 by adding noise sampled from a normal distribution with means zero and different standard deviations.

By simulated dataset 4 (SD4), we evaluated the impacts of incomplete patient relationships embedded in single data types on integrative analyses. Two prototype datasets, i.e., SD4-D0-1 and SD4-D0-2, are constructed. SD4-D0-1 simulates 100 patients by 100 variables for simplicity, in which the first 50 patients form a cluster with the first 50 variables all one and the other 50 variables all zero. The relationships of the other 50 patients are not defined in SD4-D0-1 and the corresponding variables are all zero. In SD4-D0-2, the relationships of the first 50 patients are not defined (with all the corresponding variables zero) but the other 50 patients are defined as another cluster (with the first 50 variables all zero and the other 50 variables all one). SD4-D0-1 and SD4-D0-2 together define the complete relationship of the 100 patients. SD4-T1 and SD4-T2 are constructed from SD4-D0-1 and SD4-D0-2 respectively by adding noise sampled from a normal distribution with means zero and standard deviation q.

By simulated dataset 5 (SD5), the impacts of conflicting patient relationships embedded in different data types are examined. Two prototype datasets, i.e., SD5-D0-1 and SD5-D0-2, are constructed. SD5-D0-1 simulates 100 patients by 100 variables for simplicity, in which the first 50 patients form cluster 1 with the first 50 variables all one and the other 50 variables all zero, whereas the other 50 patients form cluster 2 with the first 50 variables all zero and the other 50 variables all one. In SD5-D0-2, the first 30 patients and the last 30 patients form a cluster and the middle 40 patients form another cluster. SD5-D0-1 and SD5-D0-2 define two clusters individually but together they define four clusters of the 100 patients. SD5-T1 and SD5-T2 are constructed from SD5-D0-1 and SD5-D0-2 respectively by adding noise sampled from a normal distribution with means zero and standard deviation q.

Real datasets generally have many noisy features that are helpless to identify disease subtypes and many patients that cannot be definitely classified to a certain disease subtype. And different disease subtypes also have different sizes. We constructed another five realistic simulation datasets by adding these properties to SD1-SD5. Based on SD1-SD5, the size of the second disease subtype was doubled, 50 unclassified patients were added, and additional features (10 times of the number of informative features) that were sampled from the normal distributions were added to each simulated datasets.

### Evaluating iBFE and other integrative methods on simulated datasets

We use three types of metrics to evaluate those factors interfering with integrative analyses and the performance of various integrative methods to overcome the interfering factors in different situations. The first type of metrics examines the intra-class consistency and inter-class discrimination of patients based on the respective features constructed by individual integrative methods. Two measures are employed: Pearson correlation coefficients and the Gaussian kernel constructed based on the Euclidean distance of the extracted features. The second type of metric examines the performance of each integrative method for disease class discovery, i.e., clustering patients into subtypes. The widely used *k*-means algorithm (implemented in Matlab 8.1) is applied 1000 times to the features extracted by each integrative method with k = 2 on SD1-4 and k = 4 on SD5. The clustering scheme with the minimum sum of point-to-centroid distances is selected as the final clustering for evaluation. Normalized mutual information between the true clusters and each clustering scheme generated by different integrative methods are calculated to demonstrate their performance [5]. The third type of metric evaluates the performance of each integrative method for predicting disease classes of patients when the disease subtypes of some patients are known. The widely used random forest algorithm [29] is used as the classifier because random forest is robust and accurate and can be applied to both linearly and nonlinearly classified situations. To reduce biases caused by over-fitting, the leave-one-out cross-validation scheme is used [30].

Three integrative analysis methods are included in the evaluation, i.e., direct concatenation [5], similarity network fusion (SNF) [5] and iBFEs. Direct concatenation is included because it is the most intuitive method to integrate various types of datasets to comprehensively characterize diseases. Inclusion of direct concatenation can obviously illustrate the impacts of those suspicious factors on integrative analyses. SNF is the state-of-the-art algorithm recently proposed for integrative analyses [5], which demonstrates excellent performance in combining multiple genomic datasets to predict subtypes and survival of various cancer patients. Especially, SNF is demonstrated to outperform other integrative methods like iCluster [31] which is based on pre-selection of genes. Direct concatenation was implemented by the matrix concatenation operation in Matlab. The Matlab code of SNF was downloaded from http://compbio.cs.toronto.edu/SNF/SNF/Software.html.

### Evaluating iBFE on the DNA methylation, mRNA expression and miRNA expression datasets of lung and kidney cancers produced by TCGA

The DNA methylation, mRNA expression and miRNA expression datasets of lung squamous cell carcinoma (106 patients) and kidney renal clear cell carcinoma (122 patients) produced by TCGA are included to evaluate the performance of iBFE on real datasets [1, 4]. These two TCGA datasets are also involved in the evaluation of performance of SNF and other integrative methods [5]. Because TCGA repository contains multiple platforms for each data type, the platform corresponding to the largest number of available individuals and describing both tumor samples and controls whenever possible was enrolled in data building. For expression data, the Broad Institute HT-HG-U133A platform was included in the lung cancer dataset, and the UNC-Illumina-Hiseq-RNASeq platform was included in the kidney cancer dataset. For miRNA expression data, the BCGSC-Illumina-GA-miRNAseq platform was included in the lung and kidney cancer datasets. For the methylation data, the JHU-USC-Human-Methylation-27 platform was included in both datasets. Patients’ clinical information was also included to evaluate the prognostic power of the proposed integrative analysis method.

Three types of metrics are used to evaluate the performance of iBFE. The first type of metrics also examines the intra-class consistency and inter-class discrimination of patients and the Pearson correlation coefficients and Euclidean distances are employed. Because the true clustering schemes are not available for these two real datasets, the second and third types of metrics used on the simulated datasets cannot be used again. We proposed an alternative measure to evaluate the performance of iBFE for disease class discovery and prediction. First, k-means is applied 1000 times to obtain the clustering scheme on each cancer dataset with k ranging from 2 to 10. Then the k-means clustering scheme that is the most stable is selected as the true subtypes of patients to calculate the leave-one-out accuracy of the iBFE features, which serves as the second type of evaluating metric. The third type metric is to examine whether the integrative analyses can identify disease subtypes that have significantly different survival probability. Although factors out of the genomic measurements may also affect survival probability, prognosis prediction based on genomic data may be helpful for clinicians.

## Results

### Factors interfering with integrative analyses highlighted by simulations

We evaluated the performance of the intuitive direct concatenation method and the state-of-the-art method SNF on each type of simulated datasets. Given the controlling parameters, the simulations were repeated 100 times, and the averages of evaluating metrics were recorded for comparison. We observed that all the five factors can interfere with integrative analyses, influencing all the metrics including intra-class consistency, inter-class discrimination and accuracy of clustering and classification.

**Fig. 2:**
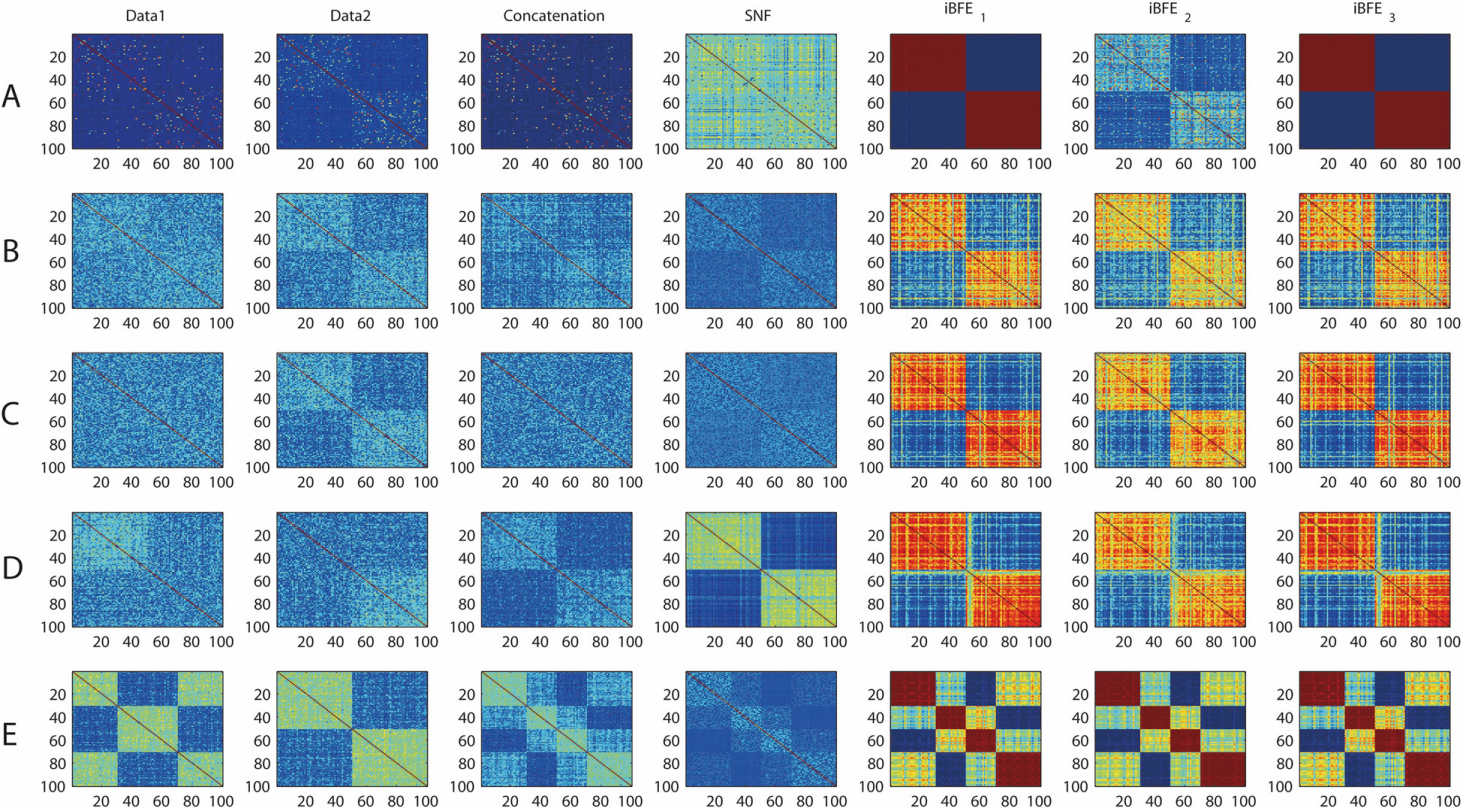
Heatmaps of patient similarity on simplistic simulation datasets. Patient similarity was measured by Pearson correlation coefficients. A, results on SD1 (issue of scales); B, results on SD2 (issue of noise types); C, results on SD3 (issue of noise sizes); D, results on SD4 (issue of incomplete patient relationships); E, results on SD5 (issue of conflict patient relationships). iBFE_1_: integration by using both Pearson and Spearman correlation coefficients; iBFE_2_: integration by using only Pearson correlation coefficients; iBFE_3_: integration by using only Spearman correlation coefficients

The different scales of two data types interfere with integrative analyses significantly when the controlling parameter q becomes large. When q is small, the scales of two data types are close to each other. And the two data types can be treated as two replicates of the same dataset. Thus, both direct concatenation and SNF can clearly identify the true patient relationships and demonstrate good performance for both class discovery and classification. However, when q is large, although direct concatenation and SNF still demonstrate acceptable discrimination of higher intra-class patient similarity than that of inter-class, the accuracy of clustering by *k*-means based on either the concatenated features or the constructed features by SNF is significantly reduced. For example, when q = 20 (Fig. 2a and Table 1), the normalized mutual information between clustering scheme produced by direct concatenation and the true patient clustering scheme is only 0.0354, whereas the normalized mutual information between clustering scheme produced by SNF and the true scheme is 0.00519. Therefore, scale issues significantly impair the accuracy of clustering based on multiple data types. For disease class prediction, direct concatenation demonstrates a good performance (94 % accuracy) when q = 20 while SNF shows dissatisfied performance (52 % accuracy).

**Table 1:** Performance comparison of different integrative analyses on simulated datasets. The average of each metric was presented and the standard deviation was not shown because the metric values are very stable between different numerical repeats

Scale issue	Data1	Data2	Concatenation	SNF	iBFE_1_	iBFE_2_	iBFE_2_
PCC_intraclass_	0.023 ± 0.0011	0.025 ± 0.0019	0.031 ± 0.0034	0.17 ± 0.0067	**0.30** ± 0.012	0.23 ± 0.014	0.28 ± 0.015
PCC_interclass_	−0.0018 ± 0.00021	−0.0067 ± 0.00033	0.0068 ± 0.00035	0.15 ± 0.0054	**−0.30** ± 0.013	−0.21 ± 0.015	−0.26 ± 0.015
PCC_intraclass_-PCC_interclass_	0.024 ± 0.0014	0.031 ± 0.0020	0.024 ± 0.0035	0.021 ± 0.012	**0.60** ± 0.022	0.44 ± 0.024	0.54 ± 0.025
Sim_intraclass_	0.02 ± 0.0023	0.02 ± 0.0022	0.02 ± 0.0023	**0.97** ± 0.004	0.32 ± 0.011	0.23 ± 0.012	0.29 ± 0.010
Sim_interclass_	**0 ± 0.0**	**0 ± 0.0**	**0 ± 0.0**	0.97 ± 0.005	0.22 ± 0.012	0.17 ± 0.013	0.22 ± 0.013
Sim_intraclass_-Sim_interclass_	0.02 ± 0.0023	0.02 ± 0.0022	0.02 ± 0.0023	0.00057 ± 0.0011	**0.10** ± 0.022	0.06 ± 0.025	0.07 ± 0.023
ACC_rfLOO	0.59 ± 0.057	0.95 ± 0.032	0.94 ± 0.045	0.52 ± 0.062	**0.99** ± 0.035	**0.99** ± 0.036	**0.99** ± 0.036
NMI_kmeans	0.035 ± 0.0085	0.035 ± 0.0089	0.035 ± 0.0093	0.0052 ± 0.0012	**0.93 ±** 0.054	0.92 ± 0.051	**0.93 ±** 0.055
Noise type							
PCC_intraclass_	0.025 ± 0.0031	0.066 ± 0.0022	0.13 ± 0.014	0.013 ± 0.0029	**0.32** ± 0.034	0.23 ± 0.030	0.31 ± 0.031
PCC_interclass_	−0.0040 ± 0.00097	−0.049 ± 0.0012	0.097 ± 0.0023	−0.011 ± 0.0058	**−0.32** ± 0.031	−0.22 ± 0.029	−0.29 ± 0.033
PCC_intraclass_-PCC_interclass_	0.029 ± 0.0032	0.12 ± 0.0024	0.033 ± 0.016	0.024 ± 0.0067	**0.64** ± 0.061	**0.45** ± 0.059	**0.60** ± 0.063
Sim_intraclass_	0.02 ± 0.0023	0.02 ± 0.0025	0.02 ± 0.0026	**0.99** ± 0.0007	0.27 ± 0.012	0.25 ± 0.015	0.28 ± 0.013
Sim_interclass_	**0** ± 0.0	**0** ± 0.0	**0** ± 0.0	0.99 ± 0.0014	0.16 ± 0.009	0.15 ± 0.011	0.16 ± 0.011
Sim_intraclass_-Sim_interclass_	0.02 ± 0.0023	0.02 ± 0.0025	0.02 ± 0.0026	0.00021 ± 0.0020	0.11 ± 0.020	0.10 ± 0.026	**0.12** ± 0.023
ACC_rfLOO	0.54 ± 0.023	**0.98** ± 0.015	0.97 ± 0.021	0.93 ± 0.013	**0.96** ± 0.015	0.95 ± 0.016	**0.96** ± 0.018
NMI_kmeans	0.015 ± 0.0021	0.82 ± 0.0023	0.024 ± 0.0033	0.0042 ± 0.00056	**0.83** ± 0.017	0.82 ± 0.018	0.82 ± 0.015
Noise size							
PCC_intraclass_	0.023 ± 0.0031	0.048 ± 0.0015	0.026 ± 0.0033	0.0070 ± 0.00067	**0.13** ± 0.038	0.09 ± 0.023	0.11 ± 0.031
PCC_interclass_	−0.0041 ± 0.00009	−0.028 ± 0.0012	−0.0071 ± 0.00013	−0.0045 ± 0.00021	**−0.13** ± 0.037	−0.08 ± 0.025	−0.10 ± 0.033
PCC_intraclass_-PCC_interclass_	0.027 ± 0.0031	0.076 ± 0.0024	0.033 ± 0.0034	0.012 ± 0.00069	**0.26** ± 0.065	0.17 ± 0.049	0.21 ± 0.061
Sim_intraclass_	0.02 ± 0.0014	0.02 ± 0.0011	0.02 ± 0.0015	**0.99** ± 0.00002	0.26 ± 0.015	0.19 ± 0.016	0.23 ± 0.017
Sim_interclass_	**0** ± 0.0	**0** ± 0.0	**0** ± 0.0	0.99 ± 0.00003	0.20 ± 0.017	0.16 ± 0.016	0.18 ± 0.018
Sim_intraclass_-Sim_interclass_	0.02 ± 0.0014	0.02 ± 0.0011	0.02 ± 0.0015	0.00016 ± 0.0006	**0.06** ± 0.027	0.03 ± 0.030	0.05 ± 0.033
ACC_rfLOO	0.59 ± 0.051	0.84 ± 0.034	0.82 ± 0.054	0.86 ± 0.041	**0.91** ± 0.052	0.90 ± 0.053	**0.91** ± 0.055
NMI_kmeans	0.024 ± 0.0081	**0.58** ± 0.043	0.028 ± 0.0097	0.0019 ± 0.00091	0.56 ± 0.062	0.55 ± 0.055	0.57 ± 0.063
Partial clustering							
PCC_intraclass_	0.049 ± 0.0038	0.046 ± 0.0042	0.06 ± 0.0021	0.027 ± 0.0023	**0.13** ± 0.05	0.10 ± 0.04	0.12 ± 0.06
PCC_interclass_	0.0010 ± 0.00085	−0.0011 ± 0.00092	−0.016 ± 0.0034	−0.025 ± 0.0026	−0.11 ± 0.023	−0.10 ± 0.024	**−0.12** ± 0.025
PCC_intraclass_-PCC_interclass_	0.048 ± 0.0043	0.048 ± 0.0047	0.079 ± 0.0070	0.053 ± 0.0049	**0.24** ± 0.067	0.20 ± 0.062	**0.24** ± 0.073
Sim_intraclass_	0.02 ± 0.0041	0.02 ± 0.0044	0.02 ± 0.0063	**0.99** ± 0.00011	0.27 ± 0.019	0.23 ± 0.020	0.25 ± 0.022
Sim_interclass_	**0** ± 0.0	**0** ± 0.0	**0** ± 0.0	0.99 ± 0.00012	0.21 ± 0.021	0.18 ± 0.021	0.20 ± 0.021
Sim_intraclass_-Sim_interclass_	0.02 ± 0.0041	0.02 ± 0.0044	0.02 ± 0.0063	0.00040 ± 0.00022	**0.061** ± 0.033	0.052 ± 0.035	0.057 ± 0.038
ACC_rfLOO	0.86 ± 0.028	0.87 ± 0.026	0.93 ± 0.016	**0.96** ± 0.029	0.90 ± 0.023	0.89 ± 0.031	0.91 ± 0.035
NMI_kmeans	0.65 ± 0.033	0.61 ± 0.032	**0.90** ± 0.028	0.63 ± 0.035	0.57 ± 0.031	0.55 ± 0.035	0.59 ± 0.039
Conflicting clustering							
PCC_intraclass_	0.095 ± 0.0052	0.095 ± 0.0051	0.095 ± 0.0061	0.032 ± 0.0063	**0.62** ± 0.023	0.53 ± 0.033	0.59 ± 0.035
PCC_interclass_	−0.017 ± 0.0047	−0.020 ± 0.0049	−0.019 ± 0.0062	−0.0099 ± 0.0067	**−0.20** ± 0.019	−0.18 ± 0.021	−0.19 ± 0.022
PCC_intraclass_-PCC_interclass_	0.11 ± 0.011	0.12 ± 0.012	0.11 ± 0.013	0.042 ± 0.015	**0.82** ± 0.037	**0.71** ± 0.045	**0.78** ± 0.051
Sim_intraclass_	0.038 ± 0.0061	0.038 ± 0.0062	0.038 ± 0.0061	**0.99** ± 0.00005	0.31 ± 0.024	0.27 ± 0.025	0.30 ± 0.029
Sim_interclass_	**0** ± 0.0	**0** ± 0.0	**0** ± 0.0	0.99 ± 0.00007	0.11 ± 0.021	0.10 ± 0.020	0.12 ± 0.023
Sim_intraclass_-Sim_interclass_	0.038 ± 0.0061	0.038 ± 0.0062	0.038 ± 0.0061	0.00019 ± 0.00010	**0.20** ± 0.037	0.17 ± 0.039	0.18 ± 0.042
ACC_rfLOO	0.42 ± 0.020	0.51 ± 0.023	0.63 ± 0.031	0.93 ± 0.063	**0.96** ± 0.034	0.94 ± 0.031	0.95 ± 0.036
NMI_kmeans	0.46 ± 0.033	0.49371 ± 0.034	0.84626 ± 0.045	0.11654 ± 0.081	**0.92** ± 0.052	**0.91** ± 0.053	**0.92** ± 0.055

The best performer was highlighted with the darkest color

PCC_intraclass_: average Pearson correlation coefficients of patients within the same classes; PCC_interclass_: average Pearson correlation coefficients of patients from different classes; Sim_intraclass_: average similarity of patients within the same classes measured by the Gausian kernel;Sim_interclass_:average similarity of patients from different classes measured by the Gausian kernel;ACC_rfLOO: accuracy of leave-one-out cross-validation by random forest; NMI_kmeans: normalized mutual information between the true patient relationships and the clustering results by k-means

The noise types and sizes also influence the integration of different data. Direct concatenation generally produces worse clustering and classification results than those based on single data (Fig. 2b and c and Table 1). Although SNF can sometimes improve the classification accuracy in leave-one-out cross-validation, the accuracy of clustering is significantly reduced (Table 1).

When the complete patient relationships are defined only by the combination of different data types and individual data type reveals only partial information of patient relationships, it is demonstrated that direct concatenation can significantly improve the intra-class consistency, the inter-class discrimination, and the accuracy of clustering and classification (Fig. 2d and Table 1). SNF also performed well with this situation, with the accuracy of classification slightly better than that of direct concatenation. However, the clustering accuracy of SNF is much lower than that of direct concatenation (Fig. 2d and Table 1).

When the patient relationships are conflictingly defined by the different data types, patients are in fact clustered to more than one class. For example, in SD5 (Fig. 2e), data1 defines two classes and data2 also defines two classes. However, the two clustering schemes are conflicting and in fact the patients form four distinct classes. The performance of direct concatenation is affected in this situation, with both the accuracy of clustering and classification reduced significantly (Fig. 2e and Table 1). In particular, the leave-one-out accuracy of classification is reduced to unsatisfied 63 %. SNF can obtain better classification accuracy (93 %) but the clustering accuracy is unsatisfied. The normalized mutual information between the true clustering scheme and the SNF clustering results became as low as to 0.12 (Fig. 2e and Table 1). Therefore, conflicting patient relationships defined by different data types impair the performance of both direct concatenation and SNF.

In summary, the performance of direct concatenation seems to be resistant to the incompleteness of patient relationships of individual data types, but it can be heavily affected by the discrepancy of scales, noise types, noise sizes, and the conflicts of the patient relationships. SNF significantly improves the classification accuracy in the situations of incomplete and conflicting patient relationships, but its clustering performance is heavily affected by these factors.

### Performance of iBFE on simulated datasets

**Fig. 3:**
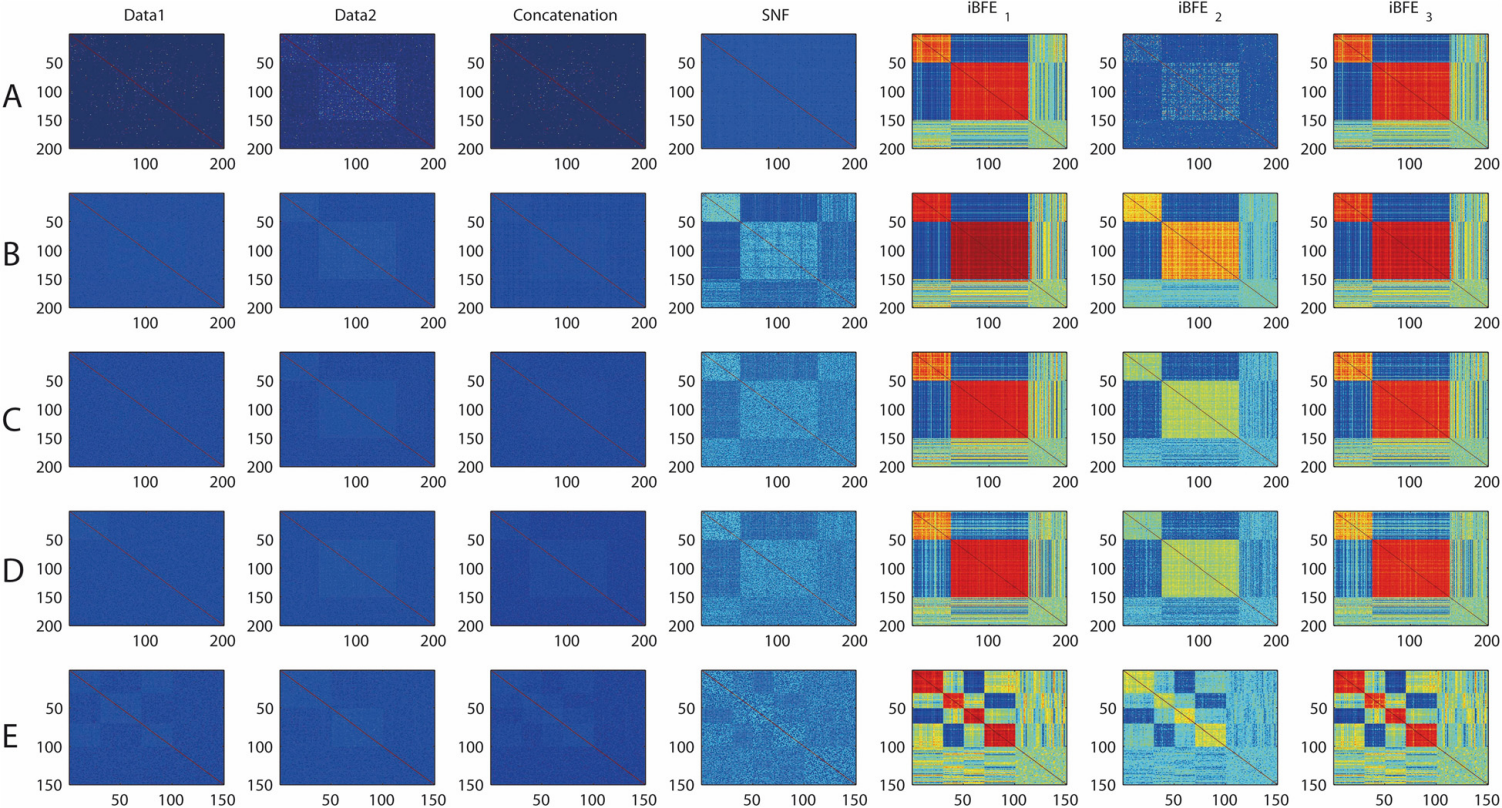
Heatmaps of patient similarity on realistic simulation datasets. Compared to simplistic simulations, realistic simulations added many noisy features and unclassified patients and the class sizes were also equal. Patient similarity was measured by Pearson correlation coefficients. A, results on SD1 (issue of scales); B, results on SD2 (issue of noise types); C, results on SD3 (issue of noise sizes); D, results on SD4 (issue of incomplete patient relationships); E, results on SD5 (issue of conflict patient relationships). iBFE1: integration by using both Pearson and Spearman correlation coefficients; iBFE2: integration by using only Pearson correlation coefficients; iBFE3: integration by using only Spearman correlation coefficients

We then applied iBFEs to the simulated datasets to evaluate whether iBFE can surmount these disturbing factors. On SD1, i.e., datasets that simulate scale issues, iBFE_1_ achieves better results than direct concatenation and SNF, regarding all the evaluation metrics including intra-class consistency, inter-class discrimination and accuracy of clustering and classification (Fig. 2a and Table 1). The leave-one-out classification accuracy of iBFE_1_ is comparable to or better than direct concatenation, and the clustering accuracy of iBFE_1_ also approximates to 1, significantly higher than those of direct concatenation and SNF. On SD2 and SD3, i.e., datasets that simulate different noise types and sizes, iBFE_1_ also outperforms direct concatenation and SNF regarding almost all the evaluation metrics (Fig. 2b and c and Table 1). On SD4 that simulates incomplete patient relationships, iBFE_1_ demonstrated better intra-class consistency and inter-class discrimination but the accuracy of clustering and classification is slightly lower than those of direct concatenation and SNF (Fig. 2d and Table 1). On SD5 that simulate conflicting patient relationships, iBFE_1_ outperformed direct concatenation and SNF regarding almost all the metrics (Fig. 2e and Table 1). On those realistic simulation datasets, iBFE_1_ also demonstrated superior performance (Fig. 3). iBFE_2_ that uses only Pearson correlation coefficients and iBFE_3_ that uses only Spearman correlation coefficients also demonstrated similar performance compared to iBFE_1_ that uses both Pearson and Spearman correlation coefficients (Figs. 2 and 3 and Table 1). Because iBFE_1_ uses more information than iBFE2 and iBFE3, it is generally more robust and often gives out clearer patterns of patient relationship (Table 1). Therefore, iBFE surmounts all the difficulties caused by the five factors regarding almost all the evaluating metrics, and it significantly outperforms direct concatenation and SNF on situations with discrepancy of scale, noise and subtype definitions.

### Performance of iBFE on real lung and kidney cancer datasets

**Fig. 4:**
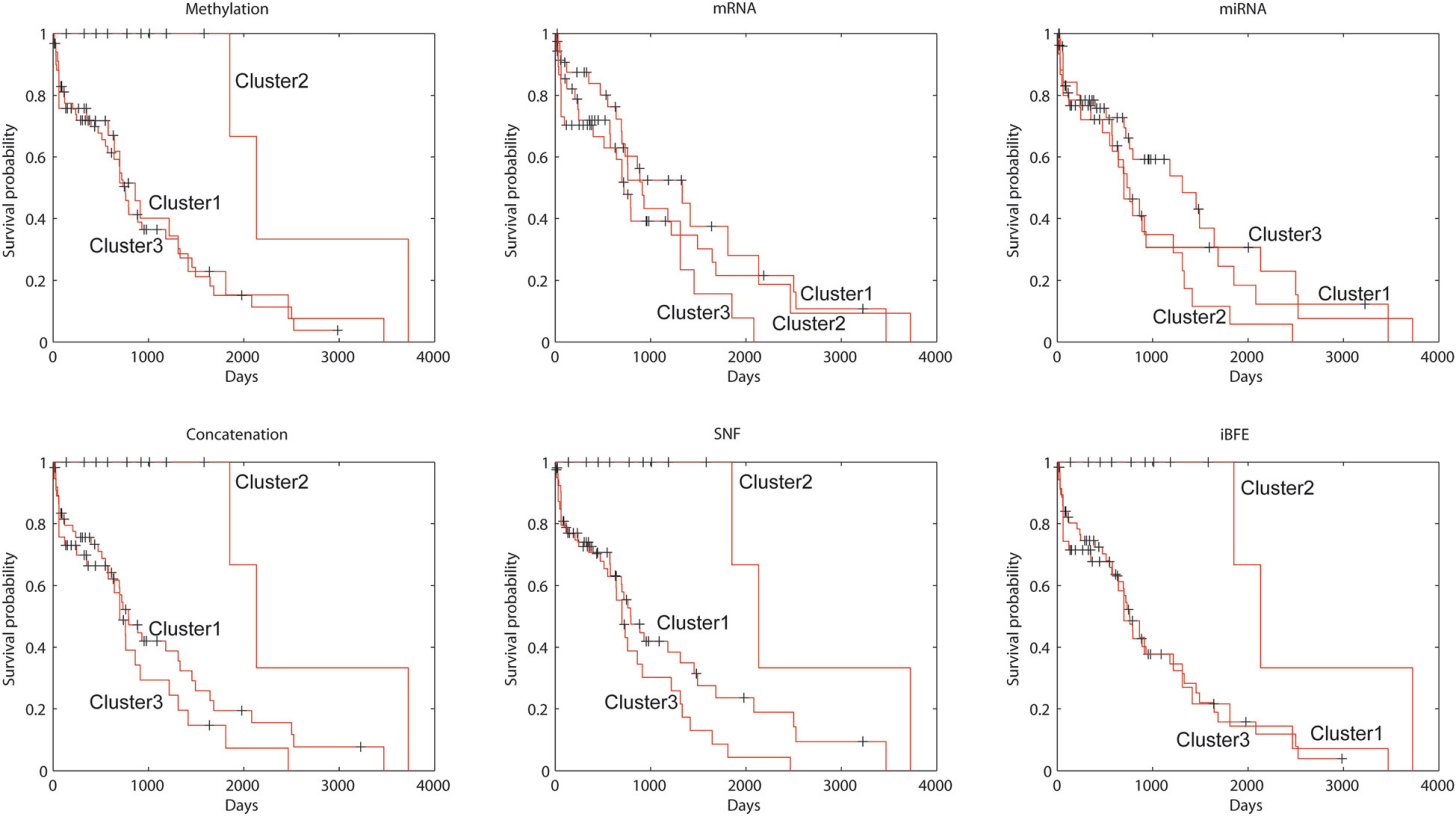
Survival curves of lung cancer subtypes revealed by different data types and integration methods

The performance of iBFE was further evaluated on real lung and kidney cancer datasets produced by TCGA. Similar to the results on simulated datasets, iBFE also demonstrated superior intra-class consistency and inter-class discrimination on both the lung and kidney cancer datasets (Fig. 4, Table 2 and Additional file 1 and Additional file 2). Based on individual clustering schemes, direct concatenation, SNF and iBFE all achieved accuracy close to 1 (Table 2).

**Table 2:** Performance comparison of different integrative methods on lung and kidney cancer datasets produced by TCGA

Lung	Methylation	mRNA	miRNA	Concatenation	SNF	iBFE
PCC_intraclass_	0.20	0.10	0.11	0.15	0.12	**0.65**
PCC_interclass_	−0.13	−0.05	−0.06	−0.10	−0.06	**−0.39**
PCC_intraclass_-PCC_interclass_	0.33	0.15	0.17	0.25	0.17	**1.04**
Dist_intraclass_	160.41	142.37	22.57	218.65	**0.02**	2.81
Dist_interclass_	239.13	157.70	26.40	**287.85**	0.02	6.29
Dist_interclass_/Dist_intraclass_	1.49	1.11	1.17	1.32	1.18	**2.24**
ACC_rfLOO	0.99	0.97	0.97	**1.00**	0.98	**1.00**
Kidney						
PCC_intraclass_	0.10	0.15	0.11	0.07	0.16	**0.37**
PCC_interclass_	−0.05	−0.07	−0.05	−0.04	−0.12	**−0.19**
PCC_intraclass_-PCC_interclass_	0.15	0.22	0.16	0.11	0.27	**0.56**
Dist_intraclass_	207.37	164.80	22.90	277.16	**0.02**	3.48
Dist_interclass_	226.39	193.05	25.64	**295.57**	0.02	4.97
Dist_interclass_/Dist_intraclass_	1.09	1.17	1.12	1.07	**1.52**	1.43
ACC_rfLOO	0.98	0.98	0.96	0.93	**1.00**	0.95

The best performer was highlighted with the darkest color

PCC_intraclass_: average Pearson correlation coefficients of patients within the same classes; PCC_interclass_: average Pearson correlation coefficients of patients from different classes; Dist_intraclass_: average Euclidean distance of patients within the same classes;Dist_interclass_:average Euclidean distance of patients from different classes;ACC_rfLOO: accuracy of leave-one-out cross-validation by random forest based on the clustering labels by *k*-means

Of the 106 lung cancer patients, 12 patients were identified to form a single cluster by all the three methods (See Additional file 1). Survival analysis demonstrated that these 12 patients showed significantly better prognosis than other patients (p = 0.00255, log-rank test for Kaplan-Meier survival functions). Within the other 94 patients, no methods identified clusters that have significantly different survival probability. This observation suggested that the performance of direct concatenation, SNF and iBFE is consistent when the signal/noise ratio is adequately high in the datasets. The discrimination of patients with better prognostics was mainly contributed by the DNA methylation data because clustering based on only methylation data also generated the same result but clustering based on mRNA expression or miRNA expression data did not obtain similar results. The normalized mutual information between clustering schemes generated by individual data types and integrative methods suggested that iBFE extracted more information from the DNA methylation data than direct concatenation and SNF.

**Fig. 5:**
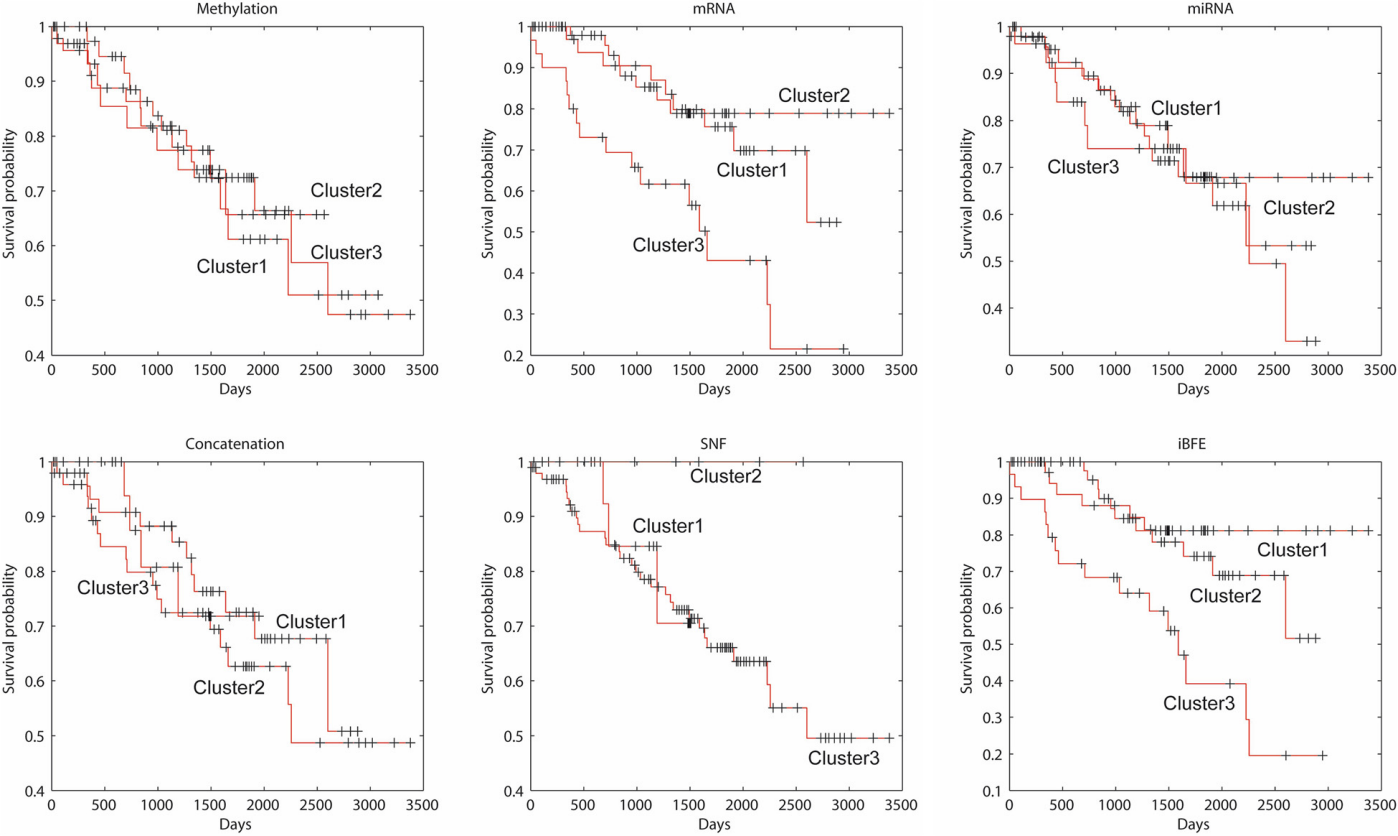
Survival curves of kidney cancer subtypes revealed by different data types and integration methods

Of the 122 kidney cancer patients, either direct concatenation or SNF did not identify patient clusters that showed significantly different prognosis. However, through clustering all the patients into three classes (so did direct concatenation and SNF), iBFE identified two classes of patients that had significantly good (*p* = 0.00892, log-rank test for Kaplan-Meier survival functions) or poor (*p* = 0.00017, log-rank test for Kaplan-Meier survival functions) prognosis against other patients (Fig. 5). The mRNA expression data contributed mainly to the identification of patient clusters with good or poor prognosis. The mRNA expression data individually suggested the existence of patient clusters with good or poor prognosis but the p-values ((*p* = 0.02109 for good prognosis and *p* = 0.00042 for poor prognosis, log-rank test for Kaplan-Meier survival functions) were higher than those of iBFE. The miRNA expression data individually identified a cluster with poor prognosis with high *p*-value (0.03033). The DNA methylation data individually did not identify clusters with significantly different prognosis. The normalized mutual information between clustering schemes generated by individual data types and integrative methods suggested that iBFE extracted more information from the mRNA expression data than direct concatenation and SNF. These results suggest that iBFE can identify and merge the signals embedded in diverse data types to accurately identify disease subtypes and predict prognosis.

## Discussion

The rapid developments of high-throughput biomedical technologies have made it possible and cost-effective to comprehensively characterize patients with various diseases from multiple levels [1, 2, 4, 5, 10, 14]. This will greatly advance the development of personalized medicine and makes hopeful promises for accurate diagnosis and prognosis [5, 10, 17, 31]. However, the heterogeneity behind the biological processes involved in the measurements and the distinct technologies also raise significant challenges for the integrative analyses [5]. Although direct concatenation is the simplest and the most intuitive method to adopt and some alternative methods have been proposed, the performance of these methods is not satisfactory and factors that hamper their performance are unclear. In this study, we dissected the possible disturbing factors and evaluated their impacts on integrative analyses by simulation, which clearly illustrate those restricting factors. Inspired by the simulation results and the fact that disease class discovery and prediction can often obtain better results in the feature space extracted from the original data [18–20], we proposed a novel method, called iBFE, for integrating diverse genomic data types towards accurately diagnosis and prognosis. Evaluation on both simulated and real datasets suggests that iBFE can overcome those restricting factors successfully. IBFE can identify patient clusters that show significantly different prognosis, which is important for understanding the subtypes of diseases and for improving patients’ health.

The principles behind iBFE are simple. Upon the feature extraction concept, iBFE employs Pearson and Spearman correlation coefficients as the atomic operations to subvert the difficulties posed by discrepancy of scales, noise and embedded patient relationships. Because Pearson correlation coefficients and Spearman correlation coefficients have no parameters to tune, iBFE is also parameter-free. Furthermore, because of the simplicity, iBFE is flexible to include other feature extraction to further improve the integrative analysis. . The same as direct concatenation and SNF, iBFE is also unsupervised. The usage of iBFE does not require any prior information of the datasets and patients. And moreover, iBFE improves the computing efficacy by transforming the original data of thousands variables into a small number of variables All these properties of iBFE greatly facilitate the application of iBFE in practice.

## Conclusions

In conclusion, we evaluated those restricting factors that hamper integrative analyses of diverse genomic datasets generated by various biomedical technologies, and proposed a simple, flexible and powerful method to overcome these restricting factors. Examinations on both simulated and real datasets suggest that the new method can effectively and efficiently identify disease subtypes and predict prognosis.

## Consent

Written informed consent was obtained from the patient by the TCGA project for the publication of this report and any accompanying images.

## Additional files

Additional file 1:
**Heatmaps of patient similarity for lung cancer.** The similarity scores were measured by the Pearson correlation coefficients based on single data types (DNA methylation, mRNA expression and miRNA expression) and the integrated scores (integrated by direct concatenation, SNF and iBFE). (DOCX 802 kb) Additional file 2:
**Heatmaps of patient similarity for kidney cancer.** The similarity scores were measured by the Pearson correlation coefficients based on single data types (DNA methylation, mRNA expression and miRNA expression) and the integrated scores (integrated by direct concatenation, SNF and iBFE). (DOCX 875 kb)

## Abbreviations

SD: Simulated dataset
SNF: Similarity network fusion
iBFE: Integration by feature extraction
TCGA: The cancer genome atlas
