# Supplementary material for: Integrating heterogeneous genomic data to accurately identify disease subtypes
Source: BMC Med Genomics. 2015 Nov 20;8:78. doi: 10.1186/s12920-015-0154-5 (PMC4653838; doi:10.1186/s12920-015-0154-5)
Supplement: Additional file 1: — Heatmaps of patient similarity for lung cancer. The similarity scores were measured by the Pearson correlation coefficients based on single data types (DNA methylation, mRNA expression and miRNA expression) and the integrated scores (integrated by direct concatenation, SNF and iBFE). (DOCX 802 kb) [file 12920_2015_154_MOESM1_ESM.docx]

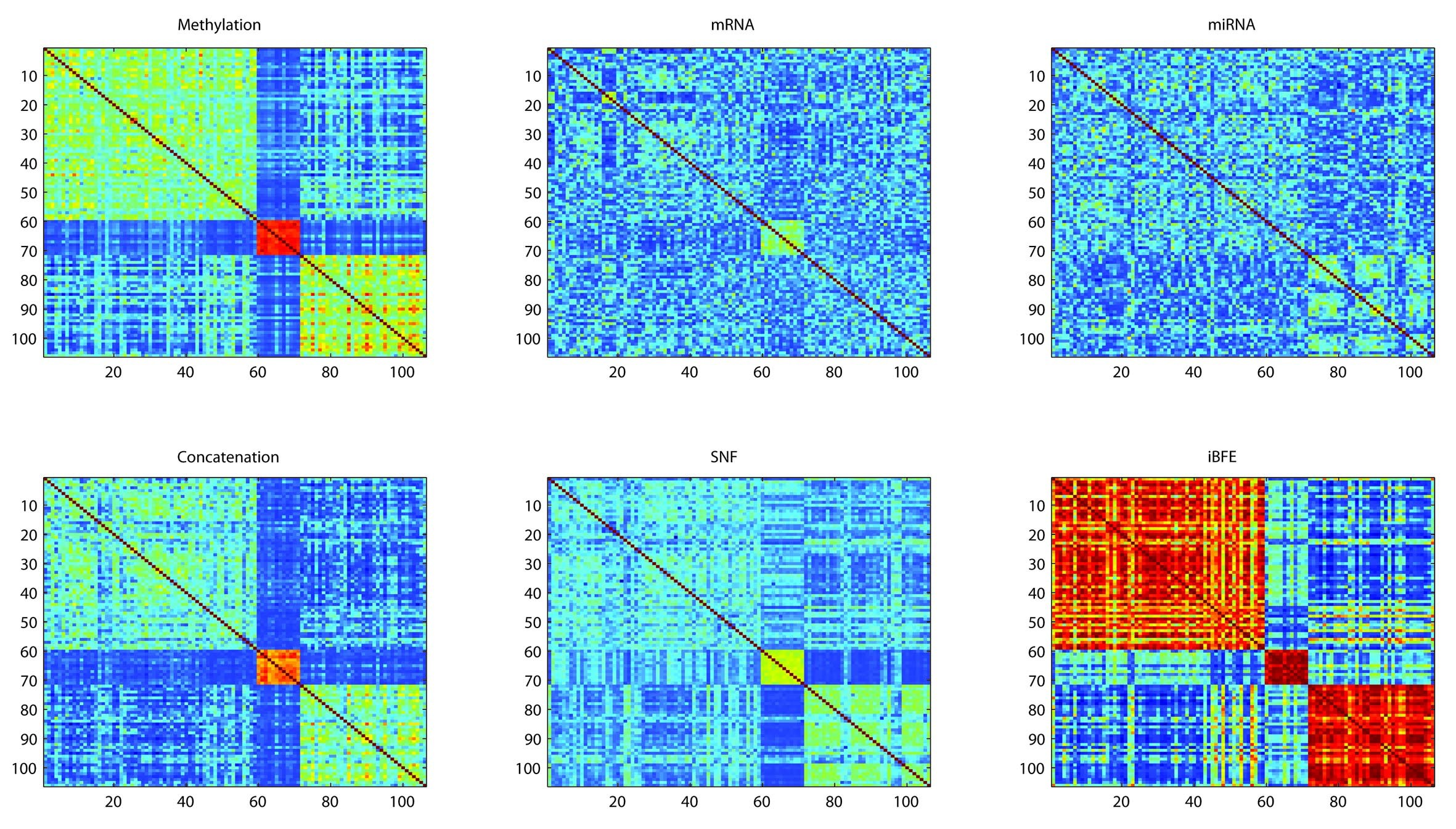


**Additional Figure 1. Heatmaps of patient similarity for lung cancer.** The similarity scores were measured by the Pearson correlation coefficients based on single data types (DNA methylation, mRNA expression and miRNA expression) and the integrated scores (integrated by direct concatenation, SNF and iBFE).
